# Isolation, identification and characteristics of *Aeromonas sobria* from diseased rainbow trout (*Oncorhynchus mykiss*)

**DOI:** 10.3389/fmicb.2024.1499126

**Published:** 2025-01-07

**Authors:** Li-Ping Liu, Ying-Dong Fang, Peng-Tian Kang, Xiang-Yun Gao, Guo-Wei Zhang, Jing Pan, Jia Lu, Ji-Xing Liu, Wang-Dong Zhang

**Affiliations:** ^1^College of Veterinary Medicine, Gansu Agricultural University, Lanzhou, China; ^2^Lanzhou Witsen Biotechnology Co., LTD, Lanzhou, China; ^3^Department of Disease Control, Gansu Fishery Technology Extension Station, Lanzhou, China

**Keywords:** *16S rRNA gene*, *gyrB* gene, histopathology, virulence genes, growth characteristics, antibiotic sensitivity

## Abstract

*Aeromonas sobria* is an opportunistic pathogen that can infect humans, animals and aquatic species, which is widely distributed in different aquatic environments and products. In recent years, with the rapid expansion of intensive aquaculture, the disease caused by *A. sobria* has occurred. This study aims to understand the pathogenic characteristics of *A. sobria* and provide scientific basis for the prevention and control of the epidemic. The dominant strain As012 was isolated from the diseased rainbow trout during the outbreak. Through physiological and biochemical experiments, sequencing and phylogenetic tree analysis of *16S rRNA* and *gyrB* genes, the strain As012 was identified as *A*. sobria. The clinical signs of the diseased rainbow trout in the experimental infection were consistent with those in the farm, and the LD_50_ was 1.0 × 10^6.6^ CFU/mL. The histopathological lesions in the gills, heart, liver, spleen and intestines were mainly extensive hemorrhage. In addition, eight virulence genes were screened from strain As012, including *Act, Aer, AexT4, Alt, ahyB, ascV, Nuc* and *Hly*. The strain As012 can grow in the environment with pH 1–11, temperature 8–43°C and NaCl concentration 0–8%. The drug sensitivity results showed that it was resistant to 12 antibiotics including penicillin G, vancomycin, and clindamycin, and highly sensitive to 16 antibiotics including cefazolin, ciprofloxacin, and furadantin. The results showed that *A. sobria*, the dominant strain isolated from diseased rainbow trout, was the main pathogen causing the epidemic in the farm. The strain As012 has a very wide range of growth and strong pathogenicity, causing widespread hemorrhaging in various tissues of rainbow trout. It is multi-resistant, but highly sensitive to cephalosporins, quinolones, nitrofurans and sulfonamides. Among them, ciprofloxacin will be one of the effective antibiotics for preventing and controlling *A. sobria* infection in Chinese aquaculture.

## 1 Introduction

The rainbow trout (*Oncorhynchus mykiss*) belongs to the order *Salmoniformes*, family *Salmonidae*, and genus *Oncorhynchus*. It is native to North America and has become one of the most widely farmed cold-water fish species in the world (Walter Devaa et al., 2023). At present, rainbow trout has been farmed in China for 65 years (Guo et al., 2016), and is favored by consumers for its fast growth, delicious meat, and rich nutrition. According to the relevant data from the Food and Aquaculture Organization of the United Nations (FAO), the world production of rainbow trout increased from 848,100 tons to 7.524 million tons during the period 2010–2018 (FAO, 2020). In recent years, with the gradual deterioration of the global water quality (Macêdo et al., 2021) and the continuous expansion of intensive aquaculture scale (Naderi et al., 2017; da Costa et al., 2019), bacterial diseases of rainbow trout have been continuously occurring. Among them, *Aeromonas hydrophila* (Cao et al., 2020) and *Aeromonas salmonicida* (Liu et al., 2022) are common pathogens in rainbow trout, while there are few reports of *A. sobria* infection in this aquatic fish.

*A. sobria* belongs to family *Aeromonadaceae* and genus *Aeromonas*. It is a Gram-negative, single-flagellated, rod-shaped, facultative anaerobic bacterium that prefers moderate temperatures (Popoff and Lallier, 1984; Yang et al., 2017). It is widely distributed in different aquatic environments and aquatic products, and is an opportunistic pathogen that can co-infect humans, livestock and aquaticanimals (Mesías et al., 2020; Majeed et al., 2023). In human reports, infection with *A. sobria* can cause meningitis, fasciitis, gastroenteritis, bacteremia, and even death due to sepsis in severe cases (Spadaro et al., 2014; Parras et al., 1993; Gelbart et al., 1985). In aquaculture, *A. sobria* can infect various aquatic animals, including tilapia (*Oreochromis niloticus*) (Li and Cai, 2011; Yang et al., 2021), African catfish (*Clarias gariepinus*) (Abdel Rahman et al., 2023), sea bass (*Dicentrarchus labrax*) (Soliman, 2022), Koi Carp (*Cyprinus carpio*) (Byadgi et al., 2018), grass carp (*Ctenopharyngodon idella*) (Mayrhofer et al., 2020), loach (*Misgurnus mizolepis*) (Yu et al., 2015) and bullfrog (*Rana catesbeiana*) (Yang et al., 2017), causing major symptoms such as skin ulcers, tail rot, fin corrosion, ascites, and hemorrhagic septicemia. Among them, the incidence rate of *A. sobria* infection in carp is 100% (Han et al., 2005) and the mortality rate is 66.67% (Abdel Rahman et al., 2022), which has caused huge economic losses to the aquaculture industry. Studies have shown that the clinical signs of specific bacterial isolates are different in different fish (Chen et al., 2019). However, the clinical signs and pathogenicity of *A. sobria* infected rainbow trout are still unknown.

Bacterial pathogens often invade host cells and cause host diseases or even death through adhesion, invasion, proliferation *in vivo* and toxin production, which involves the secretion and expression of multiple virulence factors (Qian et al., 2021). Among them, virulence factors in *Aeromonas* include aerolysin (*Aer*), cytotoxic enterotoxin (*Act, Alt, Ast*), aggregation substance (*Asa*), polar flagella (*Fla*), lateral flagella (*LafA*), hemolysin (*hly*), lipase (*Lip*), serine protease (*Ser*), elastase (*ahyB, ela*), cholesterol acyltransferase (*gcaT*), nuclease (*Nuc*), DNase (*exu*), Type III secretion system (*AscV, AexT4, aopP*), etc. (Sen and Rodgers, 2004; Nam and Joh, 2007; Nawaz et al., 2010; Burr and Frey, 2007; Alwan et al., 2022; Chacón et al., 2004). However, the secretion and expression of virulence factors are often closely related to the growth characteristics of strains (De Silva et al., 2018). Up to now, there is still a lack of relevant research on virulence factors, growth characteristics, and histopathological characteristics of *A. sobria*.

As we all know, drug susceptibility testing is an important method to evaluate the sensitivity of bacteria to antibiotics, and is also the scientific basis for effective treatment of bacterial diseases. Studies on the drug sensitivity of bacteria in the genus *Aeromonas* showed that *A. hydrophila* derived from the Siberian sturgeon (*Acipenser baerii*) was sensitive to quinolones, aminoglycosides, nitrofurans, chloramphenicol, and tetracyclines (Bakiyev et al., 2022); *A. veronii* isolated from the Crucian Carp (*Carassius auratus gibelio*) is sensitive to aminoglycosides, carbapenems, and nitrofurans (Chen et al., 2019); *A. caviae* from the source of Largemouth Bass (*Micropterus salmoides*) is highly sensitive to enrofloxacin, norfloxacin, streptomycin, and amikacin (Xue et al., 2022). However, current research on the antibiotic susceptibility of *A. sobria* is limited. Among them, the *A. sobria* derived from tilapia has multi-resistance to commonly used antibiotics (Li and Cai, 2011). However, the sensitivity of *A. sobria* isolated from rainbow trout to antibiotics is still unclear.

In mid-June 2023, the dominant strain As012 was isolated from diseased rainbow trout during the epidemic. A large number of experiments were performed, including physiological and biochemical identification, sequencing analysis and phylogenetic tree construction of *16S rRNA* and *gyrB* genes, experimental infection, screening of virulence gene, observation of growth characteristics, and antimicrobial susceptibility testing. The purpose is to identify the pathogenic bacteria of this outbreak and provide scientific basis for prevention and control of the epidemic, and lay a foundation for the study of the pathogenic characteristics and mechanism of isolate As012.

## 2 Materials and methods

### 2.1 The situation of the farm during the outbreak of the epidemic

A rainbow trout farm in Gansu Province mainly raised rainbow trout and a small amount of Gansu golden trout in cages. In mid-June 2023, during the late stage of the large-scale melting of ice and snow, a large number of rainbow trout in the farm suddenly died. Among them, the death occurred in rainbow trout weighing about 15 g, 30 g and 300 g, and in Gansu golden trout weighing about 300 g, but mainly in rainbow trout weighing 15 g and 30 g. The large fish that died had significant bleeding in the fins and tail, while the small fish that died did not have obvious bleeding characteristics, and some even had no visible lesions on their body surface. Ultimately, the mortality rate of rainbow trout in the farm reached 65%. In order to determine the pathogen of this outbreak, thirty diseased rainbow trout with obvious symptoms were randomly selected and quickly brought back to the laboratory.

### 2.2 Isolation of pathogens and observation of clinical signs of diseased rainbow trout

#### 2.2.1 Parasitological evaluation

Three rainbow trout were randomly selected and their muscles, hearts, livers, kidneys, spleens and brains were collected and examined carefully for parasites under an optical microscope (CX23, Olympus, Japan).

#### 2.2.2 Viral evaluation

Refer to the diagnosis of infectious hematopoietic necrosis in the national standards of the People's Republic of China (GB/T 15805.2-2017). The liver, kidney and spleen of 5 rainbow trout were taken as one sample, and a total of 3 samples were collected. After homogenization, approximately 50 mg of each sample was taken for viral RNA extraction. The remaining samples were diluted with DMEM medium (Gibco, China) containing 1% penicillin and streptomycin, and incubated at 15°C for 2 h. Then, the supernatant of the diluted solution was inoculated into a monolayer of carp epithelioma cells (EPC) and cultured at 15°C for 2 weeks. At the same time, viral RNA was extracted from the homogenized tissue using a viral RNA extraction kit (TaKaRa, Japan) and reverse transcribed into cDNA. Next, using cDNA as template, PCR amplification was performed based on specific primers for the infectious hematopoietic necrosis virus (IHNV) G gene (F: 5′-AGAGATCCCTACACCAGAGAC-3′ and R: 5′-TTGCACGGAAACAACACCACCA-3′, 693 bp) and the infectious pancreatic necrosis virus (IPNV) VP2 gene (F: 5′-GTGCTGGCCACAAACGACAAC-3′ and R: 5′-AATTGGTCTGCCGTTCCTA-3′, 599 bp) (Lopez-Jimena et al., 2010).

#### 2.2.3 Isolation of pathogenic bacteria and observation of clinical signs

under sterile conditions, three rainbow trout with typical clinical signs were disinfected with 75% alcohol and then dissected to observe the lesions. Meanwhile, the lesions and coelomic cavities of liver, kidney and spleen were dipped with sterile inoculating loops and streaked on the Luria-Bertani (LB) medium plate. The plates were incubated at 18°C for 32 h. Then, based on the morphological characteristics of the colonies, different colonies were selected and purified for three times. Finally, the bacterial solution was mixed with glycerol at 6:4 (v:v) and stored at −80°C.

### 2.3 Identification of strain As012

#### 2.3.1 Identification of physiological and biochemical characteristics

The physiological and biochemical characteristics of strain As012 were determined using microbiochemical reaction tube (Hangzhou Binhe Microbial Reagent Co., LTD., China). These included arginine decarboxylase, ornithine decarboxylase, lysine decarboxylase, amino acid control, citrate, nitrate reduction, urea, esculin, peptone water, β-galactoside (ONPG), sucrose, maltose, xylose, hydrogen sulfide (H_2_S), glucose, arginine dihydrolase, lactose, mannose, wood alcohol, trehalose, ethylene glycol, N-acetylglucosamine, meliobiose, fructose, phosphate-glucose peptone water, sorbitol and phenylalanine deaminase. At the same time, the purified strain As012 was cultured on starch-agar plate at 18°C for 32 h, and then the changes of the colony were observed by drops of iodine solution. Among them, the standard strain of *A. sobria* (ATCC 43979) was used as a control. The results were observed after incubation according to the manufacturer's instructions.

#### 2.3.2 Identification of molecular biology

The genomic DNA of strain As012 obtained by bacterial DNA extraction kit (TIANGEN, China) was used as template for PCR amplification based on specific primers of *16S rRNA* gene (27F: 5′-AGAGTTTGATCATGGCTCAG-3′ and 1492R: 5′-GGTTACCTTGTTACGACTT-3′) (Borrell et al., 1997) and *gyrB* gene (3F: 5′-TCCGGCGGTCTGCACGGCGT-3′ and 14R: 5′-TTGTCCGGGTTGTACTCGTC-3′) (Yáñez et al., 2003). The PCR reaction system was Premix Taq™ (TaKaRa, Japan) 12.5 μL, template DNA 1 μL, upstream and downstream primers each 1 μL, and ddH_2_O 9.5 μL. The PCR reaction conditions were predenaturation at 94°C for 5 min, 35 cycles of amplification with denaturation at 94°C for 1 min, annealing at 60°C (*16S rRNA* gene) or 55°C (*gyrB* gene) for 30 s, extension at 72°C for 2 min; final extension at 72°C for 5 min and then store at 4°C. After the PCR products were detected to be qualified by 1.5% agarose gel electrophoresis, they were sent to Jinweizhi Biotechnology Co., Ltd. (Tianjin, China) for sequencing using the ABI 3730 sequencing platform. The sequencing results were spliced and compared by BLAST, and the phylogenetic trees of isolate As012 were constructed using MEGA 11.0 software.

### 2.4 Experimental infection

Eighty healthy rainbow trout (weight 30 ± 6 g) were provided by the Cold Water Fish Breeding Center in Jingtai County, Gansu Province (Baiyin, China). They were kept in circulating water at 15 ± 1°C and fed with commercial fish food (Shandong Hanye Biotechnology Co., Ltd.) at 9:00 and 17:30 every day. First, two rainbow trout that had been domesticated for two weeks were randomly selected and their livers, kidneys, and spleens were taken for bacterial isolation. Then, under the condition of determining the absence of pathogenic bacteria infection, the rainbow trout were randomly divided into six groups, with 10 fish in each group. Meanwhile, strain As012 cultured at 18°C for 24 h was washed three times with 0.85% physiological saline and resuspended. The concentration of bacterial suspension measured by plate counting method was 1.0 × 10^9^ CFU/mL. Subsequently, 1 mL of the original bacterial suspension was taken, and the concentrations of bacterial suspension obtained by gradient dilution method were 1.0 × 10^8^, 1.0 × 10^7^, 1.0 × 10^6^ and 1.0 × 10^5^ CFU/mL in sequence. Next, rainbow trout in five experimental groups were injected with different concentrations of bacterial solution through the base of their pectoral fins (200 μL/fish), while the control group was injected with the same volume of physiological saline. Finally, the number of rainbow trout deaths was counted at 17:00 every day and observed continuously for 7 days. In addition, bacteria were isolated again from the liver, spleen, kidney and coelomic cavity of experimentally infected rainbow trout and identified according to the method in Part 1.3.2. All experimental procedures were conducted in strict accordance with the guidelines of the Animal Experimental Ethics Committee of Gansu Agricultural University.

### 2.5 Histopathological observation

The gills, heart, liver, spleen and intestinal tissues of naturally dead rainbow trout were fixed in 10% neutral formalin solution for 15 days. The paraffin sections (4 μm) were prepared according to conventional methods. After HE staining, the sections were scanned using a digital microtome scanner (DX1), and the SlideViewer software was used to select the field of view.

### 2.6 Screening of virulence genes

The virulence genes of strain As012 were screened based on the virulence genes reported in the genus *Aeromonas*. Among them, 18 virulence genes were *Act, Aer, Alt, Asa, Ast, Ela, Hly, Fla, Lip, Nuc, Ser, AexT4, ahyB, aopP, ascV, gcaT, exu* and *LafB*. The specific information of all primers is shown in Table 1. The reaction system of PCR amplification was carried out according to Part 1.3.2. Each PCR reaction commenced with predenaturation at 94°C for 5 min, then 35 cycles of amplification, and finally extension at 72°C for 5 min. Each cycle included denaturation at 94°C for 1 min, annealing for 30 s and extension at 72°C for 2 min. The annealing temperatures were performed according to Tm values in Table 1. The PCR products were detected by 1.5% agarose gel electrophoresis.

**Table 1:** Primers for PCR amplification of virulence genes.

**Gene name**	**Primer sequence (5^′^-3^′^)**	**Tm (°C)**	**Size (bp)**	**References**
*Act*	F: AGAAGGTGACCACCAAGAACA	55	232	Nawaz et al., 2010
	R: AACTGACATCGGCCTTGAACTC			
*Aer*	F: GAGCGAGAAGGTGACCACCAAGAAC	60	417	Nam and Joh, 2007
	R:TTCCAGTCCCACCACTTCACTTCAC			
*Alt*	F: TGACCCAGTCCTGGCACGGC	66	442	Sen and Rodgers, 2004
	R: GGTGATCGATCACCACCAGC			
*Asa*	F: CTGGAACCCGACTCCTTCAGC	60	143	Alwan et al., 2022
	R: CAGTTGGTGGCCTTGTCGTAC			
*Ast*	F: TCTCCATGCTTCCCTTCCACT	56	331	Sen and Rodgers, 2004
	R: GTGTAGGGATTGAAGAAGCCG			
*Ela*	F: ACACGGTCAAGGAGATCAAC	55	513	Sen and Rodgers, 2004
	R: CGCTGGTGTTGGCCAGCAGG			
*Hly*	F: GGCGGCGCCGGACGAGACGGG	62	597	Heuzenroeder et al., 199
	R: GCAGAACCCATCTATCCAG			
*Fla*	F: ATGATGGCATCTCCGTGGC	57	511	Accession number MN748929.1
	R: TGTACCGCACCGAGTTCAGC			
*Lip*	F: ATCTTCTCCGACTGGTTCGG	62	382	Sen and Rodgers, 2004
	R: CCGTGCCAGGACTGGGTCTT			
*Nuc*	F: CAGGATCTGAACCGCCTCTATCAGG	64	504	Nam and Joh, 2007
	R: GTCCCAAGCTTCGAACAGTTTACGC			
*Ser*	F: CACCGAAGTATTGGGTCAGG	57	350	Nawaz et al., 2010
	R: GGCTCATGCGTAACTCTGGT			
*AexT4*	F: GGCGCTTGGGCTCTACAC	55	553	Burr and Frey, 2007
	R: GAGCCCGCGACTCTTCAG			
*ahyB*	F: ACACGGTCAAGGAGATCAA	59	513	Nawaz et al., 2010
	R: CGCTGGTGTTGGCCAGCAGG			
*aopP*	F: GAGAGTTGGCTAGCGGTGAG	58	490	Xue et al., 2022
	R: TCCTCATGGAGCGACTCCAG			
*ascV*	F: ATGGACGGCGCCATGAAGTT	55	710	Chacón et al., 2004
	R: TATTCGCCTTCACCCATCCC			
*gcaT*	F: CTCCTGGAATCCCAAGTATCAG	65	237	Nawaz et al., 2010
	R: GGCAGGTTGAACAGCAGTATCT			
*exu*	F: (A/G)GACATGCACAACCTCTTCC	61	323	Nawaz et al., 2010
	R: GATTGGTATTGCC(C/T)TGCAA(C/G)			
*LafB*	F: GACCAGCAAGGATAGTGGGTTGGAG	64	624	Nam and Joh, 2007
	R: AAGCACCATCGCGTTGGTATAAGG			

### 2.7 Growth characteristics

When strain As012 was cultured in LB medium until the OD_600*nm*_ value reached 1.5, the bacterial solution was added into the new LB medium at a ratio of 1‰ (v:v) and cultured at 200 rpm. The absorbance value (OD_600*nm*_) under different temperature, pH and NaCl concentration was determined. Among them, the effect of temperature on the growth of strain As012 were assessed at 8°C, 13°C, 18°C, 23°C, 28°C, 33°C, 38°C, 43°C, and 48°C in LB medium with pH value of 7 and NaCl concentration of 1%. The impact of pH on the growth was investigated in LB medium at 18°C, 1% NaCl concentration and pH values of 1, 3, 5, 7, 9, 11 and 13. The effects of NaCl concentrations on the growth were evaluated in LB medium with temperature of 18°C, pH of 7 and NaCl concentration of 0–8%.

### 2.8 Antimicrobial susceptibility testing

The sensitivity of strain As012 to antibiotics was tested using the disk diffusion method (Hangzhou Binhe Microbial Reagents Co., LTD., China), which contained 35 antibiotics. According to the instructions provided by the manufacturer, 100 uL bacterial solution with a concentration of 1 × 10^5^ CFU/mL was evenly spread on the LB medium plate, and then the drug-sensitive papers were affixed to the plate using sterile tweezers. Among them, each antibiotic pasted 3 drug-sensitive papers. Next, after incubating the plates at 18°C for 24 h, the diameters of the inhibition zones for each antibiotic were measured. Finally, the average diameter of the inhibition zones of the three drug-sensitive papers was used as the antibacterial result of the antibiotic. The sensitivity of strain As012 to antibiotics was determined based on the range of the inhibition zone set by the manufacturer.

## 3 Results

### 3.1 Isolation of pathogens and observation of clinical signs of diseased rainbow trout

#### 3.1.1 Isolation of pathogenic bacteria

No parasites were found in the tissue during parasitological evaluation. During the virological evaluation, no cytopathic effect was observed in EPCs, and no target bands were detected in PCR amplification. Therefore, the rainbow trout in this farm have been ruled out of the possibility of parasitic and viral infection. During the bacteriological examination, the dominant strain isolated was temporarily named As012.

#### 3.1.2 Observation of clinical signs of diseased rainbow trout

**Figure 1:**
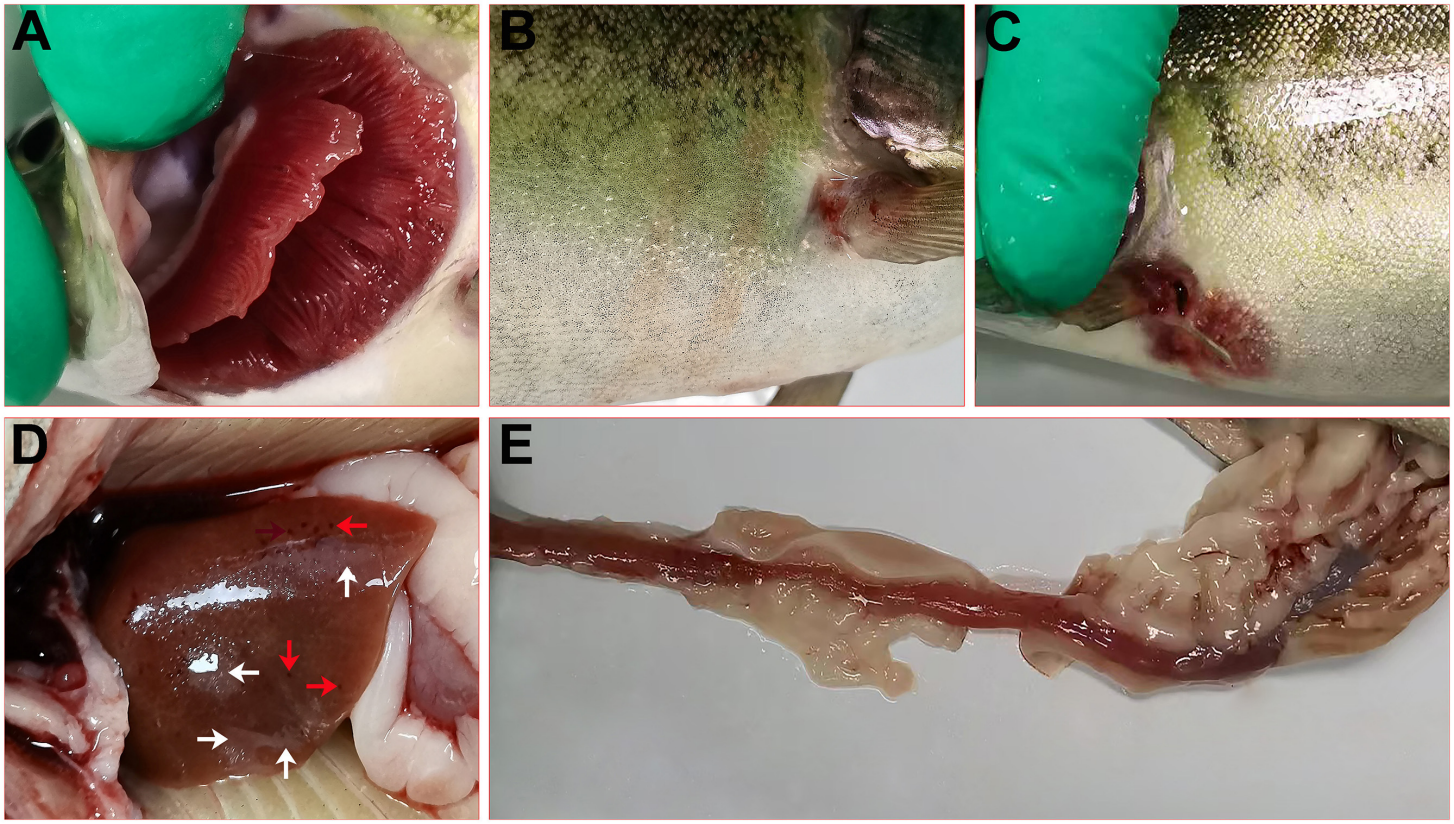
Clinical signs of diseased rainbow trout. **(A)** White gill filaments with large amounts of mucus. **(B)** Hyperemia on the body surface and swelling of the fins. **(C)** Bleeding and decay of the fins. **(D)** Bleeding (red arrow) and necrosis (white arrow) in the liver. **(E)** Intestinal congestion and hemorrhage.

The diseased rainbow trout in the farm showed obvious clinical signs, including white gill filaments with large amounts of mucus (Figure 1A), hyperemia on the body surface (Figure 1B), swelling, bleeding, and decay of the fins (Figures 1B, 1C). The liver showed punctate hemorrhage and grayish necrotic lesions (Figure 1D). The intestines were congested, hemorrhagic, and devoid of chyme (Figure 1E).

### 3.2 Identification of strain As012

#### 3.2.1 Physiological and biochemical identification

The results of physiological and biochemical tests were shown in Table 2. The strain As012 showed positive reaction in ornithine decarboxylase, lysine decarboxylase, amino acid control, urea, sucrose, maltose, xylose, glucose, lactose, mannose, wood alcohol, trehalose, ethylene glycol, N-acetylglucosamine, meliobiose, fructose, sorbitol, phenylalanine deaminase and starch. The reaction was negative in arginine decarboxylase, citrate, nitrate reduction, esculin, peptone water, β-galactoside (ONPG), H_2_S, arginine dihydrolase and phosphate-glucose peptone water. Except for phosphate-glucose peptone water and sorbitol, the remaining items were consistent with the characteristics of standard strain *A. sobria*. In addition, the results of strain As012 and standard strain *A. sobria* in xylose and starch were completely opposite to those of *A. veronii* (Chen et al., 2019). Therefore, the strain As012 was preliminarily identified as *A. sobria*.

**Table 2:** Physiological and biochemical characteristics of strain As012.

**Name**	**Reaction**			**Name**	**Reaction**		
	**As012**	***A. sobria***	***A. veronii***		**As012**	***A. sobria***	***A. veronii***
Arginine decarboxylase	-	-	/	Glucose	+	+	+
Ornithine decarboxylase	+	+	+	Arginine dihydrolase	-	-	-
Lysine decarboxylase	+	+	+	Lactose	+	+	+
Amino acid control	+	+	/	Mannose	+	+	+
Citrate	-	-	-	Wood alcohol	+	+	/
Nitrate reduction	-	-	/	Trehalose	+	+	/
Urea	+	+	+	Ethylene glycol	+	+	/
Esculin	-	-	-	N-acetylglucosamine	+	+	/
Peptone water	-	-	/	Melibiose	+	+	/
Beta-galactoside (ONPG)	-	-	/	Fructose	+	+	+
Sucrose	+	+	+	Phosphate-glucose peptone water	-	+	/
Maltose	+	+	+	Sorbitol	+	-	-
Xylose	+	+	-	Phenylalanine deaminase	+	+	/
Hydrogen sulfide (H_2_S)	-	-	-	Starch	+	+	-

+, positive; –, negative;/, unknown. *A. sobria* (ATCC 43979); *A. veronii* (Chen et al., 2019).

#### 3.2.2 Sequencing analysis of *16S rRNA* and *gyrB* genes

**Figure 2:**
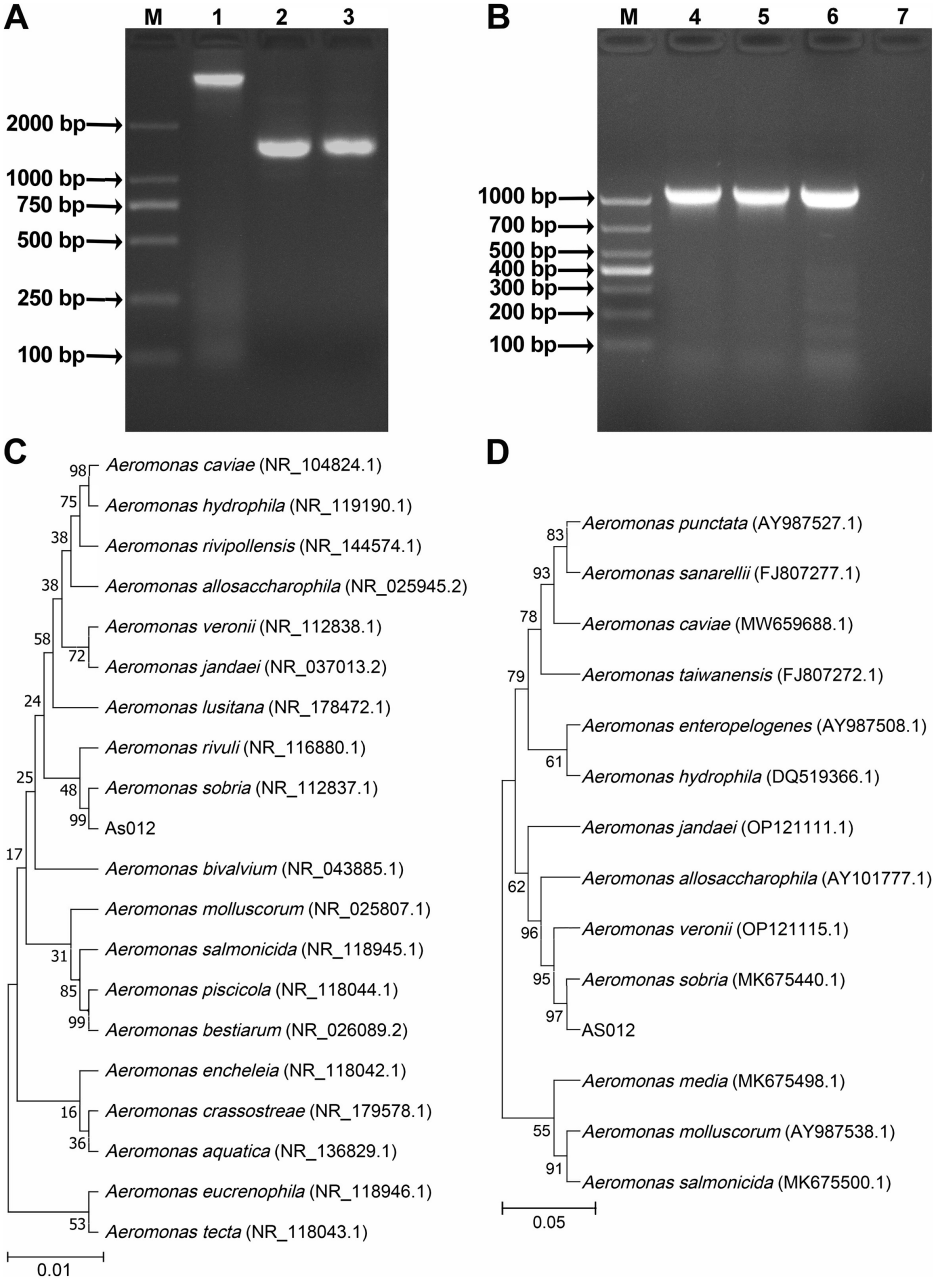
PCR amplification and phylogenetic tree construction of *16S rRNA* and *gyrB* genes. **(A)** PCR amplification results of *16S rRNA* gene. **(B)** PCR amplification results of *gyrB* gene. **(C)** Phylogenetic tree of *16S rRNA* gene sequence. **(D)** Phylogenetic tree of *gyrB* gene sequence. M: Marker; 1: Genomic DNA of strain As012; 2-3: *16S rRNA* gene; 4-6: *gyrB* gene; 7: Negative control.

The length of *16S rRNA* (GenBank accession number: PQ517010) and *gyrB* (GenBank accession number: PQ527032) gene sequences were 1417 bp and 1013 bp, respectively. PCR amplification results were shown in Figures 2A, 2B. The compared results showed that the similarity between the *16S rRNA* gene sequence with *A. sobria* NR_037012.2 was 99.86%, and the similarity between the *gyrB* gene sequence with *A. sobria* AB473159.1 was 98.41%. At the same time, phylogenetic trees were constructed using gene sequences of *16S rRNA* (Figure 2C) and *gyrB* (Figure 2D), and the results showed that strain As012 clustered with *A. sobria* in the same branch. In summary, the strain As012 was identified as *A. sobria*.

### 3.3 Experimental infection

The first death occurred in rainbow trout at 12 h after injection of *A. sobria solution*. The clinical signs of experimentally infected rainbow trout were consistent with those of similar-sized fish in the farm. In addition, the dominant strain *A. sobria* was isolated again from the liver, spleen, kidney and coelomic cavity of the experimentally infected rainbow trout, indicating that *A. sobria* was the main pathogen causing the outbreak in the farm.

**Figure 3:**
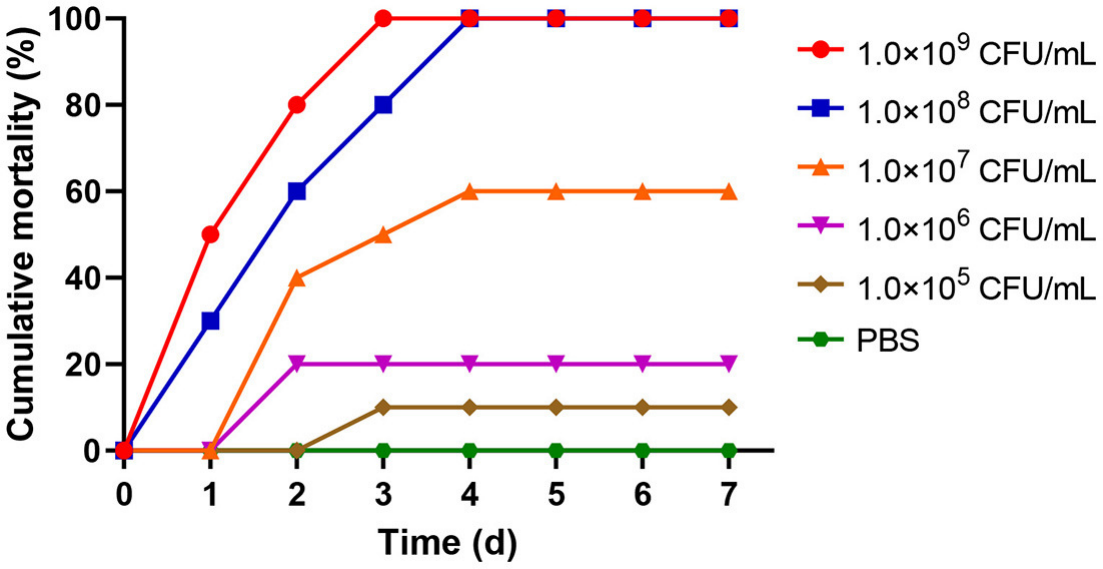
The cumulative mortality of rainbow trout infected with different concentrations of strain As012.

When rainbow trout were infected with different concentrations of *A. sobria* solution on day 7, the cumulative mortality of 1.0 × 10^9^ and 1.0 × 10^8^ CFU/mL groups was 100%. The cumulative mortality rates of the 1.0 × 10^7^, 1.0 × 10^6^ and 1.0 × 10^5^ CFU/mL groups were 60%, 20% and 10%, respectively. However, there were no deaths in the control group (Figure 3). The LD_50_ of strain As012 was calculated by the Karber method to be 1.0 × 10^6.6^ CFU/mL (Markus et al., 1995).

### 3.4 Histopathological observation of rainbow trout

**Figure 4:**
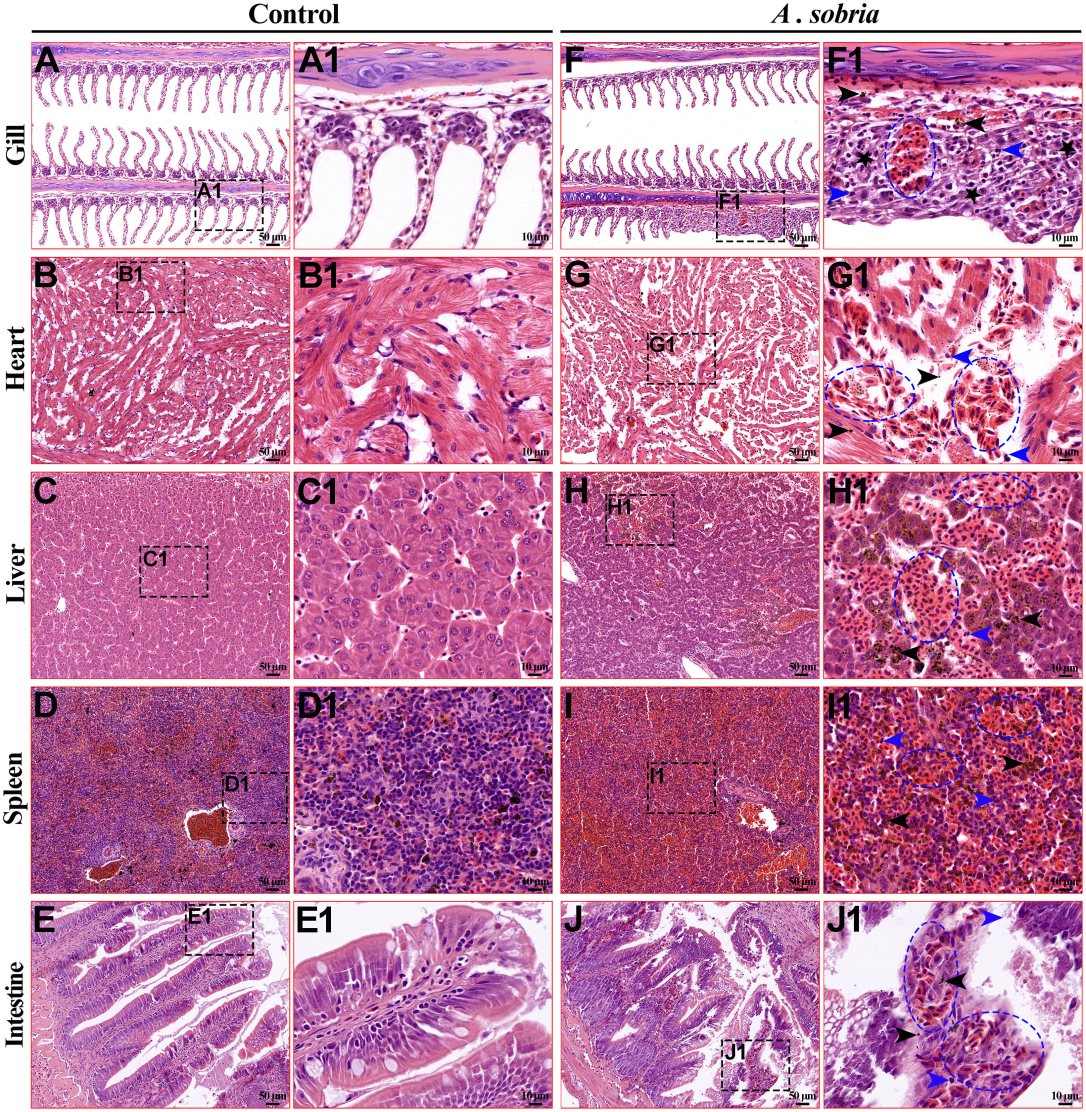
Histological lesions of the diseased rainbow trout. **(A–E)** were the gills, heart, liver, spleen and intestine of the control group, with a microscope magnification of 200x. **(F–J)** were the gills, heart, liver, spleen and intestine of the infected group, with a microscope magnification of 200x. **(A1–J1)** were the local magnified images of **(A–J)** respectively, with a microscopic magnification of 1,000x. Among them, the pathological changes of the tissue mainly include bleeding (blue ellipse), inflammatory cell infiltration (blue arrow), deposition of hemosiderin (black arrow), and hyperplasia (black pentagram).

Compared with the control group (Figures 4A, 4A1, 4B, 4B1, 4C, 4C1, 4D, 4D1, 4E, 4E1), the naturally infected rainbow trout showed obvious pathological changes. Among them, the branchial lamella became shorter, hypertrophied and hemorrhagic (Figures 4F, 4F1). There was extensive diffuse hemorrhage in the heart, liver and spleen (Figures 4G–4I), with large amounts of hemosiderin deposition and inflammatory cell infiltration (Figures 4G1, 4H1, 4I1). The intestinal villi were bleeding, damaged, and shed, with inflammatory cell infiltration between epithelial cells and vacuolation of cells in the mucosal layer (Figures 4J, 4J1). In summary, *A. sobria* infection mainly causes extensive hemorrhaging in the tissues of rainbow trout.

### 3.5 Screening results of virulence genes

**Figure 5:**
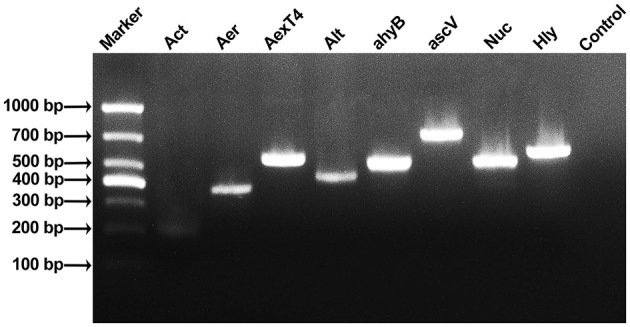
Results of PCR amplification of virulence genes of strain As012. The eight virulence genes were *Act, Aer, AexT4, Alt, ahyB, ascV, Nuc*, and *Hly*.

In this study, 18 virulence genes related to the genus *Aeromonas* were screened by PCR amplification. Eight virulence genes (*Act, Aer, AexT4, Alt, ahyB, ascV, Nuc*, and *Hly*) were amplified in strain As012, and the fragment sizes were basically consistent with the target gene fragment sizes (Figure 5).

### 3.6 Growth characteristics of strain As012

**Figure 6:**
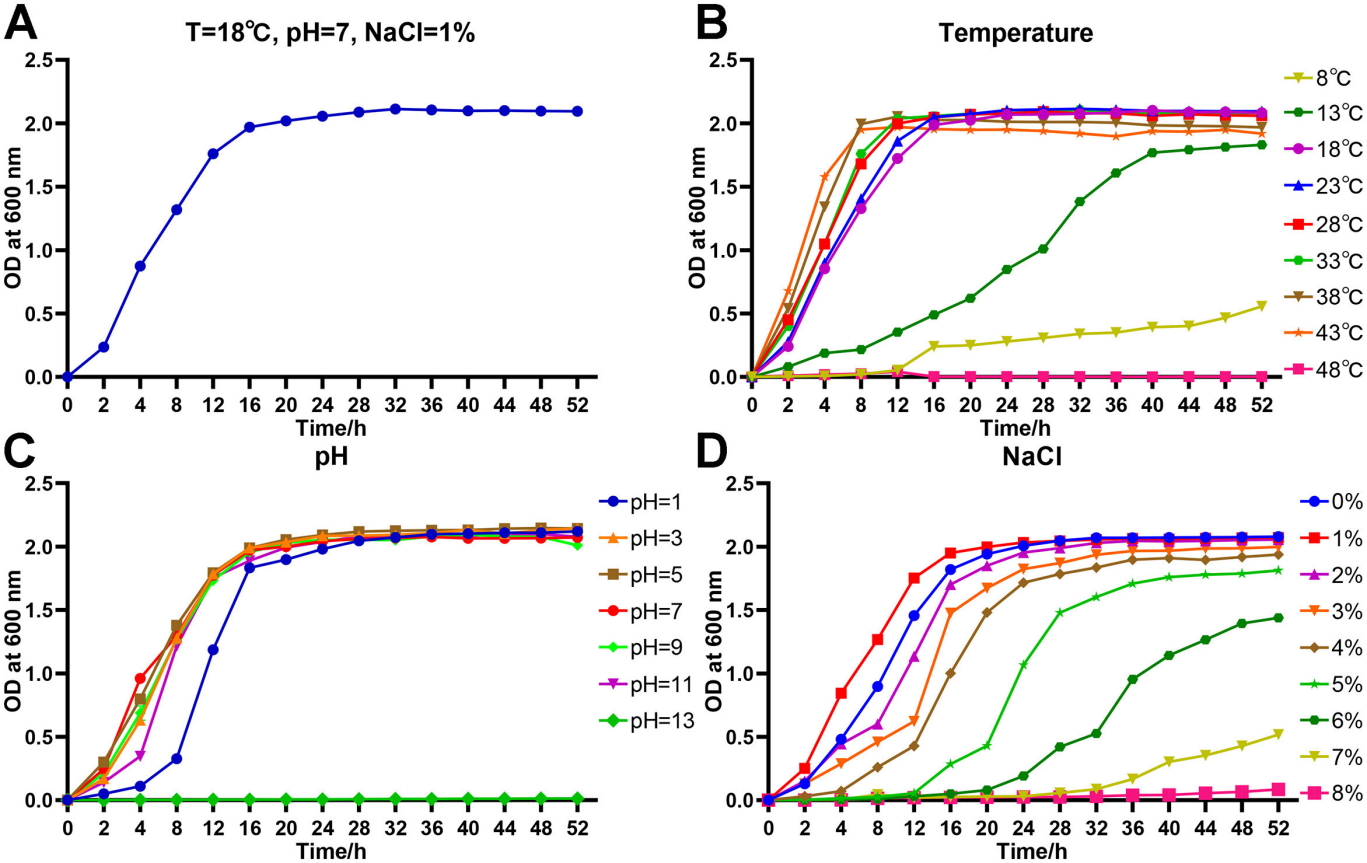
Growth characteristics of strain As012. **(A)** Growth curve under the conditions of T=18°C, pH=7, and NaCl=1%. **(B)** Growth curves at different temperatures. **(C)** Growth curves at different pH values. **(D)** Growth curves at different NaCl concentrations.

Under the conditions of T = 18°C, pH = 7 and NaCl = 1%, the growth curve of strain As012 showed that the slow growth phase was 0–2 h, the logarithmic growth phase was 2–16 h, and the bacterial growth entered the stable phase after 16 h **(**Figure 6A).

The strain As012 grew well at temperatures of 18–43°C and all entered stable growth phase after 16 h (Figure 6B). However, the strain began to enter stable growth phase with OD_600nm_ < 2.0 when growing at 13°C for 40 h. It grew slowly at 8°C and OD_600nm_ was about 0.5 at 52 h. The strain As012 grew well at pH values of 1, 3, 5, 7, 9 and 11, and all entered the stable phase after 24 h (Figure 6C). However, the growth of strain As012 was completely inhibited at pH=13.

The strain As012 was able to grow under conditions of NaCl concentrations ranging from 0% to 8% (Figure 6D). Among them, the strain As012 grew best at NaCl concentration of 1% and entered the stable phase after 16 h. Subsequently, as the NaCl concentration increased, the slow growth phase of strain As012 gradually prolonged. At the same time, under the condition of NaCl concentration of 2–6%, the time point for strain As012 to enter the stable growth phase was continuously delayed, and the OD_600nm_ value of the stable phase gradually decreased.

### 3.7 Antimicrobial susceptibility testing

The results of the antimicrobial susceptibility testing showed (Table 3) that the strain As012 was highly sensitive to four classes of antibiotics, including cephalosporins (cefazolin, cefalotin, cefuroxime, cefoperazone, cefotaxime, ceftriaxone, ceftazidime, cefepime, cefoxitin, aztreonam), quinolones (norfloxacin, ciprofloxacin, ofloxacin, levofloxacin), nitrofurans (furadantin), and sulfonamides (sulfamethoxazole). It was intermediately sensitive to piperacillin, polymyxin B, kanamycin, spectinomycin, amikacin, minocycline, chloramphenicol. And it was resistant to penicillins (penicillin G, oxacillin, ampicillin), polypeptides (vancomycin), lincoamides (clindamycin), macrolides (erythromycin, clarithromycin, midecamycin), aminoglycosides (streptomycin, tobramycin, gentamicin and tetracycline). Among them, the strain As012 was the most sensitive to cephalosporins, followed by quinolones, sulfonamides and nitrofurans.

**Table 3:** Sensitivity test of strain As012 to 35 antibiotics.

**Types of antibiotics**	**Drug name**	**Content (ug/table)**	**Judgement standard/mm**			**Diameter of inhibition zone/mm**	**Susceptibility**
			**R**	**I**	**S**		
Penicillins	Penicillin G	10	≤ 19	20–27	≥26	0	R
	Oxacillin	10	≤ 10	11–12	≥13	0	R
	Ampicillin	1	≤ 13	14–16	≥17	0	R
	Piperacillin	100	≤ 17	18–20	≥21	19 ± 0.20	I
Cephalosporins	Cefazolin	30	≤ 14	15–17	≥18	25 ± 0.03	S
	Cefalotin	30	≤ 14	15–17	≥18	23 ± 0.11	S
	Cefuroxime	30	≤ 14	15–17	≥18	26 ± 0.09	S
	Cefoperazone	75	≤ 15	16–20	≥21	30 ± 0.30	S
	Cefotaxime	30	≤ 14	15–22	≥23	35 ± 0.25	S
	Ceftriaxone	30	≤ 13	14–20	≥21	37 ± 0.16	S
	Ceftazidime	10	≤ 14	15–17	≥18	31 ± 0.19	S
	Cefepime	30	≤ 14	15–17	≥18	28 ± 0.32	S
	Cefoxitin	30	≤ 14	15–17	≥18	32 ± 0.14	S
	Aztreonam	10	≤ 15	16–21	≥22	23 ± 0.22	S
Polypeptides	Vancomycin	10	≤ 14	15–16	≥17	0	R
	Polymyxin B	30	≤ 8	9–11	≥12	11 ± 0.17	I
Lincoamides	Clindamycin	30	≤ 14	15–20	≥21	0	R
Macrolides	Erythromycin	15	≤ 13	14–22	≥23	11 ± 0.21	R
	Clarithromycin	30	≤ 13	14–17	≥18	0	R
	Midecamycin	300	≤ 13	14–17	≥18	12 ± 0.23	R
Aminoglycosides	Streptomycin	1.25	≤ 11	12–14	≥15	9 ± 0.45	R
	Kanamycin	300	≤ 13	14–17	≥18	16 ± 0.34	I
	Tobramycin	30	≤ 12	13–14	≥15	0	R
	Spectinomycin	30	≤ 14	15–17	≥18	17 ± 0.26	I
	Gentamicin	10	≤ 12	13–14	≥15	0	R
	Amikacin	100	≤ 14	15–16	≥17	16 ± 0.18	I
Tetracyclines	Tetracycline	5	≤ 14	15–18	≥19	11 ± 0.25	R
	Minocycline	5	≤ 14	15–18	≥19	16 ± 0.32	I
Chloramphenicols	Chloramphenicol	30	≤ 12	13–17	≥18	12 ± 0.44	I
Quinolones	Norfloxacin	30	≤ 12	13–16	≥17	29 ± 0.31	S
	Ciprofloxacin	30	≤ 15	16–20	≥21	27 ± 0.27	S
	Ofloxacin	15	≤ 12	13–15	≥16	29 ± 0.42	S
	Levofloxacin	5	≤ 13	14–16	≥17	32 ± 0.38	S
Nitrofurans	Furadantin	2	≤ 14	15–16	≥17	21 ± 0.50	S
Sulfonamides	Sulfamethoxazole	30	≤ 10	11–15	≥16	26 ± 0.19	S

R, Resistant; I, Intermediate susceptible; S, Susceptible.

## 4 Discussion

The reported pathogenic bacteria in genus *Aeromonas* that can cause bacterial diseases in aquatic animals include *A. hydrophila* (Guo et al., 2024), *A. veronii* (Zhu et al., 2023), *A. salmonicida* (Liu et al., 2023), *A. caviae* (Xue et al., 2022), *A. jandaei* (Chen et al., 2024), *A. sobria* (Byadgi et al., 2018), etc. The diseases caused by them in aquaculture often occur in aquatic animals with deteriorating water quality (Alwan et al., 2022), impaired organism, or low immune function (Song et al., 2023). In this study, the region was in the late stage of large-scale melting of ice and snow when the epidemic occurred. The deterioration of water quality and the rise in water temperature have led to the proliferation of pathogenic bacteria, which meets the environmental conditions for the occurrence of *Aeromonas* diseases. In addition, studies have shown that lower water temperatures will slow down the metabolic rate of fish, leading to reduced immunity (Flinders and Magoulick, 2017; Song et al., 2019; Le Morvan et al., 1998). After enduring a cold and long winter, the rainbow trout in this farm are currently in a phase of weakened immune function. At this time, in intensive farming, improper management can easily lead to the outbreak of bacterial diseases. Therefore, after excluding the possibility of parasitic and viral infections, the dominant strain As012 isolated from the diseased fish may be the main pathogen responsible for the outbreak of the epidemic in this farm.

The results of physiological and biochemical identification of strain As012 showed that it was negative for nitrate reduction and phosphate glucose peptone water, and positive for sorbitol. The remaining characteristics were completely consistent with the standard strain of *A. sobria*, but also very close to those of *A. veronii* (Chen et al., 2019). The difference is that the starch agar plate test in this study showed that strain As012 can utilize starch, which is completely consistent with previous studies (Langó et al., 2002), indicating that *A. sobria* can produce enzymes that decompose and utilize starch. However, *A. veronii* is unable to utilize starch (Chen et al., 2019). Whether this difference can be directly used as the only characteristic distinguishing *A. sobria* from *A. veronii* has not been confirmed. In this case, it is necessary to use molecular biological methods to classify and identify bacteria. Among them, *16S rRNA* gene sequencing is one of the most commonly used methods for bacterial species identification and phylogenetic analysis (Ntushelo, 2013). However, the high conservation of *16S rRNA* gene sequence leads to low discrimination among closely related species, especially when studying bacteria of the genus *Aeromonas* (Chen et al., 2019; Küpfer et al., 2006). It is documented that the *gyrB* gene is more prone to mutation without changing the amino acid sequence, and this mutation precisely makes it easier to distinguish closely related strains (Watanabe et al., 2001; Zhu et al., 2023). More and more studies have combined *16S rRNA* with *gyrB* gene sequences for species identification and phylogenetic analysis of *Salmonella* (Tajbakhsh et al., 2011), *Flavobacterium* (Peeters and Willems, 2011), *Nocardia* (Takeda et al., 2010), and *Aeromonas* (Küpfer et al., 2006), and all have achieved satisfactory results. In this study, the sequencing results were Blast compared, and the results showed that the *16S rRNA* and *gyrB* gene sequences of strain As012 had the highest homology with the known *A. sobria*. Meanwhile, phylogenetic analysis of the two sequences showed that they clustered into the same clade as *A. sobria*. Therefore, the dominant strain As012 isolated from the current outbreak was successfully identified as *A. sobria*.

Virulence factors are not only the criteria to judge the virulence and toxicity of pathogens (Jiang et al., 2023; Cepas and Soto, 2020), but also the key to reveal the mechanism of bacterial infection (Howden et al., 2023). In this study, 8 virulence genes were detected in strain As012 among the 18 virulence genes related to the genus *Aeromonas*, including *Act, Aer, AexT4, Alt, ahyB, ascV, Nuc* and *Hly*. Previous studies have detected *Act, Aer, Alt*, and *Nuc* genes from different sources of *A. sobria* (Alwan et al., 2022; Nam and Joh, 2007), suggesting that the five virulence genes present in *A. sobria* may not be affected by the source of the strain. Among them, *Aer*, a gene encoding aerolysin, was first discovered in *A. hydrophila* and plays a key role in pathogen migration and invasion of pathogens (Abrami et al., 2003). The enterotoxin genes encoded by *Act* and *Alt* are closely related to gastroenteritis and diarrhea (Albert et al., 2000). The elastase encoded by *ahyB* gene supports pathogen infection and colonization by destroying tissues and degrading immune proteins (Song et al., 2018). The hemolysin encoded by the *Hly* gene can destroy cell membranes, leading to the lysis of red blood cells, and assist bacteria to escape the host's inflammatory response (Gao et al., 2013). The *AscV* gene is a highly conserved endosomal component of the Type III secretion system (T3SS) (Burr et al., 2002). Studies have shown that the mutation or deletion of *AscV* gene will lead to the closure of secretory channels of T3SS, preventing the secretion and translocation of cytotoxins such as *AexT* and *AexU*, thereby significantly reducing the virulence of *A. hydrophila* and *A. salmonicida* (Fadl et al., 2006; Burr et al., 2005). Therefore, the presence of the *AscV* gene in this study indicates that the strain As012 has strong pathogenicity. At present, *AexT4* and *Nuc* genes have been detected in the isolates of *A. hydrophila, A. veronii* and *A. caviae* (Aravena-Román et al., 2014; Nam and Joh, 2007), but their functions are still unclear. At the same time, the synergistic effect of multiple virulence factors is the key factor leading to the high pathogenicity of pathogens to the body (Beaz-Hidalgo and Figueras, 2013; Zhao et al., 2020). Studies have shown that when *Aeromonas* carries the *Aer* and *Hly* genes, it can cause massive bleeding and hemolysis in host tissues (Xu et al., 2022) and is considered as a potential foodborne pathogen (Reyes-Rodríguez et al., 2019; Lee et al., 2023). In addition, studies have shown that strains containing both *Aer* and *Act* genes are virulent strains (Gashgari and Selim, 2015). The pathological results of this study showed that the infected rainbow trout had extensive hemorrhage in various tissues and a large amount of hemosiderin deposition in the heart, liver and spleen, which may be the result of the combined action of multiple virulence factors such as *Hly, Aer* and *Act* in *A. sobria*.

As is well known, the adaptability of bacteria to pH, temperature, salt concentration directly determines their survival and reproductive ability in different environments. This study showed that strain As012 could grow in environments with pH of 1–11, temperature of 8–43°C, and salt concentration of 0–8%, which was similar to the growth characteristics of *A. allosaccharophila* (Shao et al., 2023), *A. veronii* (Chen et al., 2019) and *A. hydrophila* (Bakiyev et al., 2022). The difference is that the strain As012 is more tolerant to acids, bases and high concentrations of salts, indicating that the growth environment of this strain is more extensive. In actual aquaculture, temperature is the key factor determining the speed of bacterial reproduction (Janda and Abbott, 2010). For example, bacterial diseases rarely occur in the cold winters, but they frequently break out in spring, summer and autumn when the environment temperature is 15–40°C. The optimal growth temperature of rainbow trout is 13–18°C (Magoulick and Wilzbach, 1998), which is suitable for the growth and reproduction of *A. sobria*. In addition, the tolerance of *A. sobria* to salt indicates that it not only affects the development of freshwater aquaculture, but may also exist in seawater and related products. Therefore, the special growth characteristics will bring great challenges for the prevention and control of *A. sobria*.

At present, antibiotics remain the most economical and effective strategy for the prevention and treatment of bacterial diseases. In this study, *A. sobria* was most sensitive to cephalosporins (cefazolin, cefalotin, cefuroxime, cefoperazone, cefotaxime, ceftriaxone, ceftazidime, cefepime, cefazolin, and aztreonam), followed by quinolones (norfloxacin, ciprofloxacin, ofloxacin, and levofloxacin), sulfonamides (sulfamethoxazole), and nitrofurans (furadantin). At present, according to the provisions of the “Aquaculture Drug Use White Paper No. 1 and No. 2 in 2022” issued by the Ministry of Agriculture and Rural Affairs of China, and combined with the drug sensitivity results of this experiment, ciprofloxacin and its related preparations can be used to effectively prevent and treat *A. sobria* in practical aquaculture. In addition, *A. sobria* was resistant to penicillins (penicillin G, oxacillin, ampicillin), polypeptides (vancomycin), lincoamides (clindamycin), macrolides (erythromycin, clarithromycin, madecycin) and aminoglycosides (streptomycin, tobramycin, gentamicin, tetracycline), indicating that this strain was a multidrug-resistant bacterium. Meanwhile, Comparing the antibiotic sensitivity results of this study with those of *A. sobria* strain PY36 from tilapia, which revealed that *A. sobria* from different animal sources have different antibiotic resistance spectra. For example, The strain As012 from rainbow trout is resistant to erythromycin, streptomycin, and tetracycline, while the strain PY36 from tilapia is highly sensitive to them (Li and Cai, 2011). This phenomenon has also been reported in *A. veronii* (Hu et al., 2023) and *A. jandaei* (Chen et al., 2024), which may be the result of continuous selection of antibiotic resistance genes in bacteria during long-term evolution due to different growth environments. Therefore, it is necessary to monitor daily antibiotic resistance in the prevention and control of bacterial diseases in aquaculture, and to use antibiotics reasonably and effectively during outbreaks of diseases.

In summary, *A. sobria*, the dominant strain isolated from the diseased rainbow trout in this study, is the main pathogen causing the outbreak in this farm. The characteristic studies showed that the strain not only has a very wide range of growth, but also has strong pathogenicity, causing extensive hemorrhaging in various tissues of rainbow trout. The strain was highly sensitive to 16 antibiotics such as cefazolin, ciprofloxacin, and furadantin; Moderately sensitive to 7 antibiotics including piperacillin, polymyxin B, kanamycin, etc.; Resistant to 12 antibiotics including penicillin G, vancomycin, and clindamycin. Among them, ciprofloxacin will be one of the preferred antibiotics for the prevention and treatment of *A. sobria* in Chinese aquaculture. This study will provide reference for the prevention and treatment of *A. sobria* diseases and lay a foundation for the study of its pathogenic mechanism.

## Data Availability

The data presented in the study are deposited in the NCBI repository, accession numbers PQ517010 and PQ527032.
